# Evidence for Induction of Integron-Based Antibiotic Resistance by the SOS Response in a Clinical Setting

**DOI:** 10.1371/journal.ppat.1002778

**Published:** 2012-06-14

**Authors:** Didier Hocquet, Catherine Llanes, Michelle Thouverez, Hemantha D. Kulasekara, Xavier Bertrand, Patrick Plésiat, Didier Mazel, Samuel I. Miller

**Affiliations:** 1 Department of Immunology, Medicine and Microbiology, University of Washington, Seattle, Washington, United States of America; 2 EA4266, Laboratoire de Bactériologie, Université de Franche-Comté, Besançon, France; 3 Laboratoire d'Hygiène Hospitalière, CHRU, Besançon, France; 4 UMR6249 Chrono-Environnement, Université de Franche-Comté, Besançon, France; 5 Institut Pasteur, Unité Plasticité du Génome Bactérien, CNRS UMR3525, Département Génomes et Génétique, Paris, France; Northwestern University, United States of America

## Abstract

Bacterial resistance to β-lactams may rely on acquired β-lactamases encoded by class 1 integron-borne genes. Rearrangement of integron cassette arrays is mediated by the integrase IntI1. It has been previously established that integrase expression can be activated by the SOS response *in vitro*, leading to speculation that this is an important clinical mechanism of acquiring resistance. Here we report the first *in vivo* evidence of the impact of SOS response activated by the antibiotic treatment given to a patient and its output in terms of resistance development. We identified a new mechanism of modulation of antibiotic resistance in integrons, based on the insertion of a genetic element, the *gcuF1* cassette, upstream of the integron-borne cassette *bla*_OXA-28_ encoding an extended spectrum β-lactamase. This insertion creates the fused protein GCUF1-OXA-28 and modulates the transcription, the translation, and the secretion of the β-lactamase in a *Pseudomonas aeruginosa* isolate (S-*Pae*) susceptible to the third generation cephalosporin ceftazidime. We found that the metronidazole, not an anti-pseudomonal antibiotic given to the first patient infected with S-*Pae*, triggered the SOS response that subsequently activated the integrase IntI1 expression. This resulted in the rearrangement of the integron gene cassette array, through excision of the *gcuF1* cassette, and the full expression the β-lactamase in an isolate (R-*Pae*) highly resistant to ceftazidime, which further spread to other patients within our hospital. Our results demonstrate that in human hosts, the antibiotic-induced SOS response in pathogens could play a pivotal role in adaptation process of the bacteria.

## Author Summary

The bacterial SOS response is a conserved regulatory network that is induced in response to DNA damage. Its activation *in vitro* leads to the emergence of resistance to antibiotics, leading to speculation that this is an important clinical mechanism of acquiring resistance. We found evidence here that antibiotic-induced SOS response plays a role in bacterial genome rearrangement *in viv*o within humans. The major classes of antibiotics can trigger the bacterial SOS response and our data raise questions about their wide use and their subsequent effect on the bacterial genetic adaptability. This suggests that emergence of antibiotic resistance during therapy could be reduced by inhibiting the bacterial SOS response. We showed that acquired resistance genes could spread latently in susceptible bacterial strains until needed. These findings could impact current policies for control of antibiotic resistance, which rely on the detection of resistant bacteria and on the assumption that resistance mechanisms have a functional cost to the bacteria. More generally, SOS response may spur changes in the behavior of bacteria and their faster adaptation to hostile environments, including humans.

## Introduction

Transferable genes encoding antibiotic resistance to major antibiotics (*e.g.* β-lactams, aminoglycosides) are often carried by class 1 integrons in Gram negative pathogens [1]. In these genetic elements the antibiotic resistance genes are carried inside mobile structures called gene cassettes, which generally correspond to a promoterless gene associated to a recombination site called *attC*, formerly called 59-be [2], [3]. Gene cassette expression is driven by a promoter located in the integron platform upstream of the *attI* site, the primary site of cassette integration, and in the case of the class 1 integron, inside the *intI1* gene which encodes the cassette recombinase [4]. This organization allows a positional regulation of the cassette's expression: the closer a gene cassette is located to *attI*, the higher is its expression [1]. In addition to this transcriptional attenuation along the cassette array, the decrease in expression can be due to problems of translational coupling [5], [6]. Thus, gene expression in these elements can be modulated by the site-specific recombination events mediated by the integrase IntI1 [1], [3].

The SOS response is a conserved regulatory network that is induced in response to DNA damage [7]. It also promotes integron rearrangements by controlling the expression of integrases with promoters that contain a LexA-binding motif [8], [9]. During the SOS response, the RecA protein, bound to single stranded DNA, stimulates the cleavage of the repressor LexA, thus releasing the transcription of the LexA-controlled genes. The adaptations resulting from integron activity are thought to influence bacterial evolution, especially in *Proteobacteria*, where integrons are extremely common [3]. We have recently shown that common horizontal gene transfer processes, such as conjugation [10], trigger the SOS response and ultimately the integron integrase expression. However, the most medically relevant SOS induction is certainly the one directed by antibiotic treatments. Hence, a number of antibiotics, including the β-lactams, aminoglycosides and fluoroquinolones, have been found to directly or indirectly provoke this stress response [11]–[13]. Despite this evidence from *in vitro* studies, the clinical significance of the SOS response on integron rearrangement and the dynamics of integron-based bacterial adaptation during human infections are unknown. So far, there are no examples of SOS-mediated antibiotic resistance occurring during therapeutic use of antibiotics, even though, as mentioned above, many of them stimulate the SOS response.

Here, we witnessed the emergence in a hospitalized patient of an isolate of *Pseudomonas aeruginosa* highly resistant to the third generation cephalosporin ceftazidime, associated with the production of an extended-spectrum β-lactamase encoded by a class 1 integron-borne gene. This strain, highly resistant to ceftazidime, further became epidemic within the hospital. We discovered the mechanism, based on the excision of a gene cassette originally located upstream of the β-lactamase-encoding gene cassette, which modulated the expression of the transferable resistance gene. This patient had been previously treated with ceftazidime (to treat the infection by *P. aeruginosa*) and metronidazole (to treat an infection by anaerobes). This led us to suspect the involvement of this treatment in the integron cassette array remodeling, through SOS response induction inside the patient. We demonstrated that the metronidazole, not an anti-pseudomonal antibiotic, is able to trigger the SOS response in *P. aeruginosa*, and subsequently activates the integrase IntI1 and cassette rearrangement. Deletion of the *gcuF1* cassette and full expression the β-lactamase were obtained at high rates *in vitro*, supporting this scenario to explain the genesis, in the patient, of the R-*Pae* isolate from S-*Pae* after metronidazole treatment.

## Results

### Enzymatic resistance mechanisms to β-lactams in an epidemic multidrug resistant *P. aeruginosa*

**Figure 1:**
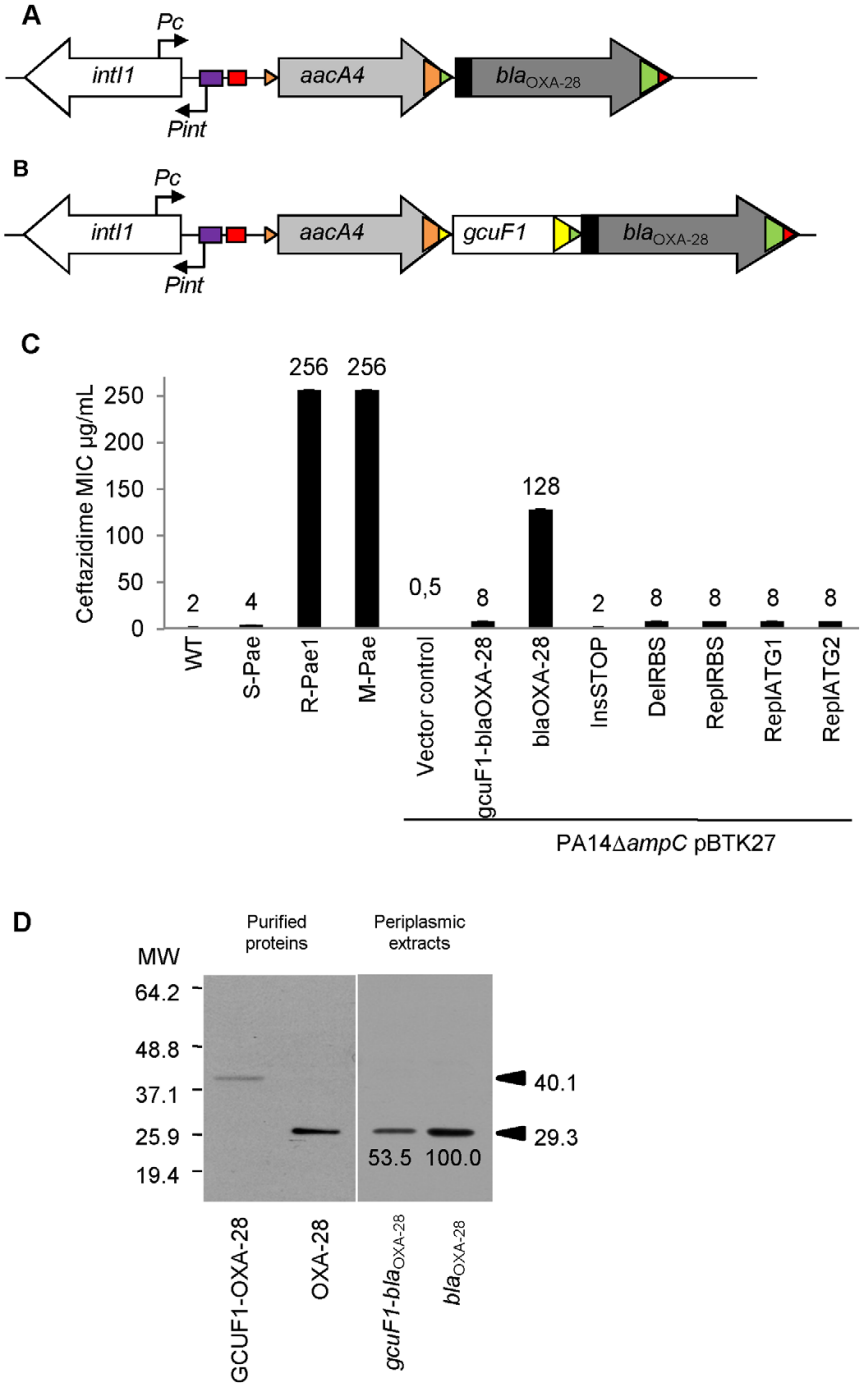
*gcuF1* cassette insertion modulates the resistance to ceftazidime. (A) Schematic representation of the class 1 integron carried by R-*Pae*1 and laboratory M-*Pae* mutants obtained from S-*Pae* and (B) carried by S-*Pae*, with the 319 bp *gcuF1* cassette. Open arrows represent the coding sequences and indicate the direction of transcription. The purple box indicates the LexA box. The *attI1* site is shown by the red box and colored triangles indicate the cassette *attC* sites. The black rectangles indicate the peptide signal sequences encoded in *bla*_OXA-28_. The genes *intI1*, *aacA4 and bla*_OXA-28_ encode the integrase IntI1, the aminoglycoside acetyltransferase AAC(6′)-Ib and the extended-spectrum oxacillinase OXA-28, respectively. The nucleotide sequences reported here appear in the EMBL/Genbank nucleotide sequence database under accession no. FJ207466 (S-*Pae*) and FJ374756 (R-*Pae*1). (C) Susceptibility to ceftazidime of *P. aeruginosa* reference strain PA14 (WT), S-*Pae*, R-*Pae*1, laboratory ceftazidime-resistant mutant obtained from S-*Pae* (M-*Pae*) and PA14Δ*ampC* carrying the empty vector pBTK27 (Vector control), cloned *gcuF1*-*bla*_OXA-28_ (*gcuF1*-*bla*_OXA-28_), cloned *bla*_OXA-28_ (*bla*_OXA-28_) and with site-directed mutations in *gcuF1*-*bla*_OXA-28_: TGA stop codon inserted downstream of *gcuF1* (InsSTOP), GAAGG ribosome binding site deleted upstream of *bla*_OXA-28_ (DelRBS) or substituted with a sequence with no translation initiation power (ReplRBS) and with the *bla*_OXA-28_ ATG start codon replaced by GTC (ReplATG1) or GTG (ReplATG2). Means ± SEM (*n* = 3). Numbers above bars are MICs of ceftazidime. (D) Left pane: Insertion of *gcuF1* creates a new ORF. SDS-PAGE analysis of purified N-terminal His-tagged GCUF1-OXA-28 and OXA-28 from *E. coli*. Right pane: Insertion of *gcuF1* reduces the amount of processed periplasmic OXA-28. SDS-PAGE analysis of periplasmic extracts (10 µg of total proteins) from reference strain *P. aeruginosa* PA14Δ*ampC* producing C-terminal His-tagged GCUF1-OXA-28 and OXA-28 (relative band intensities are indicated under the bands). Molecular weights are indicated in kDa.

**Figure 2:**
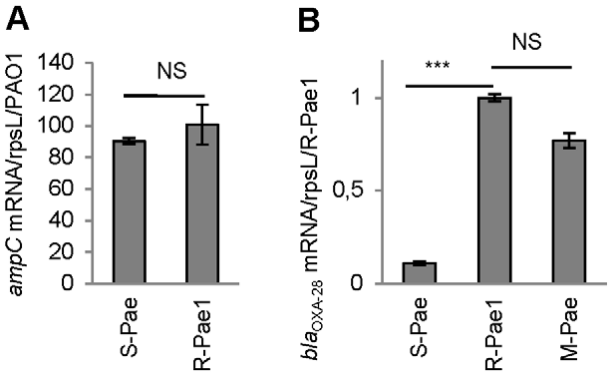
*gcuF1* excision enhances the expression of *bla*_OXA-28_. The mRNA levels of genes are given. Means ± SEM (*n*≥2, two-sided Student's *t*-test; NS, *P*>0.05; ***, *P*<0.001). Transcripts of gene *ampC* (A) and *bla*_OXA-28_ (B) were quantitatively assessed by RT-qPCR. The mRNA levels of genes were normalized to that of reference gene *rpsL* and expressed as a ratio to the levels in the R-*Pae*1 isolate (for *bla*_OXA-28_) or in the wild-type PA14 (for *ampC*) in which the values were set at 1.00.

We detected the same clone of a multi-drug resistant clone of *P. aeruginosa* (R*-Pae*) in 13 adult patients by pulsed-field gel electrophoresis (Figure S1). R*-Pae* was resistant to potent anti-pseudomonal agents, including ceftazidime, cefepime, aztreonam, aminoglycosides, and fluoroquinolones (Table S1). A double-disk synergy test revealed a weak synergy between β-lactamase substrates (ceftazidime or cefepime) and β-lactamase inhibitors (imipenem or clavulanate) in these bacteria, suggesting production of an extended-spectrum β-lactamase (oxacillinase) of Ambler class D (Figure S2) [14]. We searched for the most common genes encoding extended-spectrum oxacillinases in *P. aeruginosa* [15] by PCR for the *bl*a_OXA-10_, *bla*_OXA-2_ and *bla*_OXA-1_ groups [16]. Only the PCR specific to *bla*_OXA-10_ using primers OXA-10A and B (see Table S2) was positive. As *bla*_OXA-10_ and variants are found as gene cassettes often borne by class 1 integrons [17], we performed PCR experiments using primers specific to these integron platforms, directed to the conserved sequences flanking the variable cassette array, usually called 5′-CS and 3′-CS (see Table S2). A single 1673-bp amplicon was thus obtained, which was subsequently sequenced to reveal two resistance gene cassettes, namely *aacA4* that determines a 6′-*N*-aminoglycoside acetyltransferase conferring high resistance to gentamicin and tobramycin [18], and *bla*_OXA-28_ that encodes the extended-spectrum oxacillinase OXA-28 [19] (Figure 1A). Additionally, quantification of the specific mRNA transcripts by RT-qPCR showed that the R-*Pae*1 isolate overexpressed the *ampC* gene encoding the intrinsic chromosomal cephalosporinase AmpC, when compared to the wild type reference strain of *P. aeruginosa* PAO1 (Figure 2A).

To determine whether bacterial resistance had emerged during the course of treatment, we retroactively analyzed the *P. aeruginosa* isolates archived from the first colonized patient (patient 1) within 2 months before the isolation of R-*Pae*. The clonal strain S-*Pae* was isolated from the sputum of patient 1, 28 days before the isolation of R-*Pae* in the patient's lung. R-*Pae*1 was isolated after a treatment with ceftazidime (to treat the infection by *P. aeruginosa*) and metronidazole (to treat an infection by anaerobes). To our surprise, S-*Pae* was susceptible to ceftazidime (MIC, 4 µg/ml) despite the presence of an intact *bla*_OXA-28_ gene in its genome (Figure 1B, C). S-*Pae* and R*-Pae*1 demonstrated an equivalent expression level of the cephalosporinase-encoding *ampC* (Figure 2A). The other resistance mechanisms found in S-*Pae* and R*-Pae*1 (efflux pump overproduction and porin loss) do not alter the susceptibility to ceftazidime (see Text S1). Because of the lack of evidence for classical resistance mechanisms accounting for the difference in β-lactam susceptibilities in these isolates, we investigated the possible mechanism of resistance modulation.

### OXA-28 oxacillinase production in R-*Pae*1 and S-*Pae*

**Figure 3:**
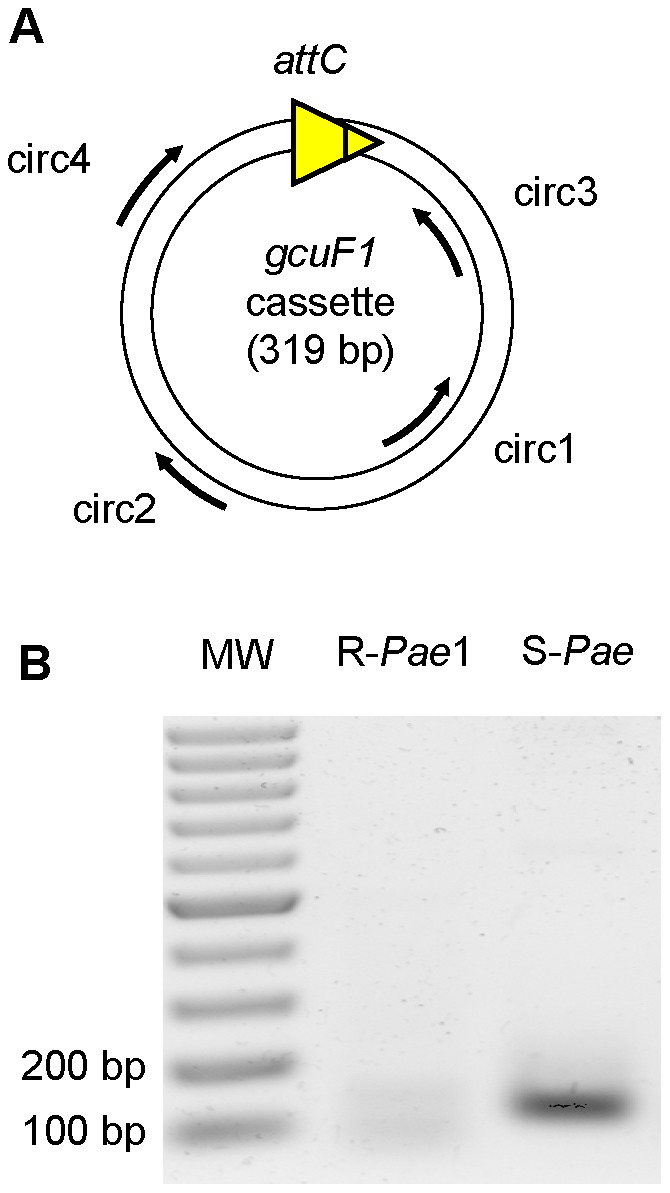
*gcuF1* gene cassette exists in free circular form. (A) Position and direction of primers used for amplification (Table S2) are indicated by arrows. (B) Visualization of the PCR products on an agarose gel (resulted from the nested PCR with primers circ3 and circ4). PCRs were performed using total DNA from isolates R-*Pae*1 (without *gcuF1*, taken as a negative control) and S-*Pae* (with *gcuF1*) as templates. MW: Molecular weight.

**Figure 4:**
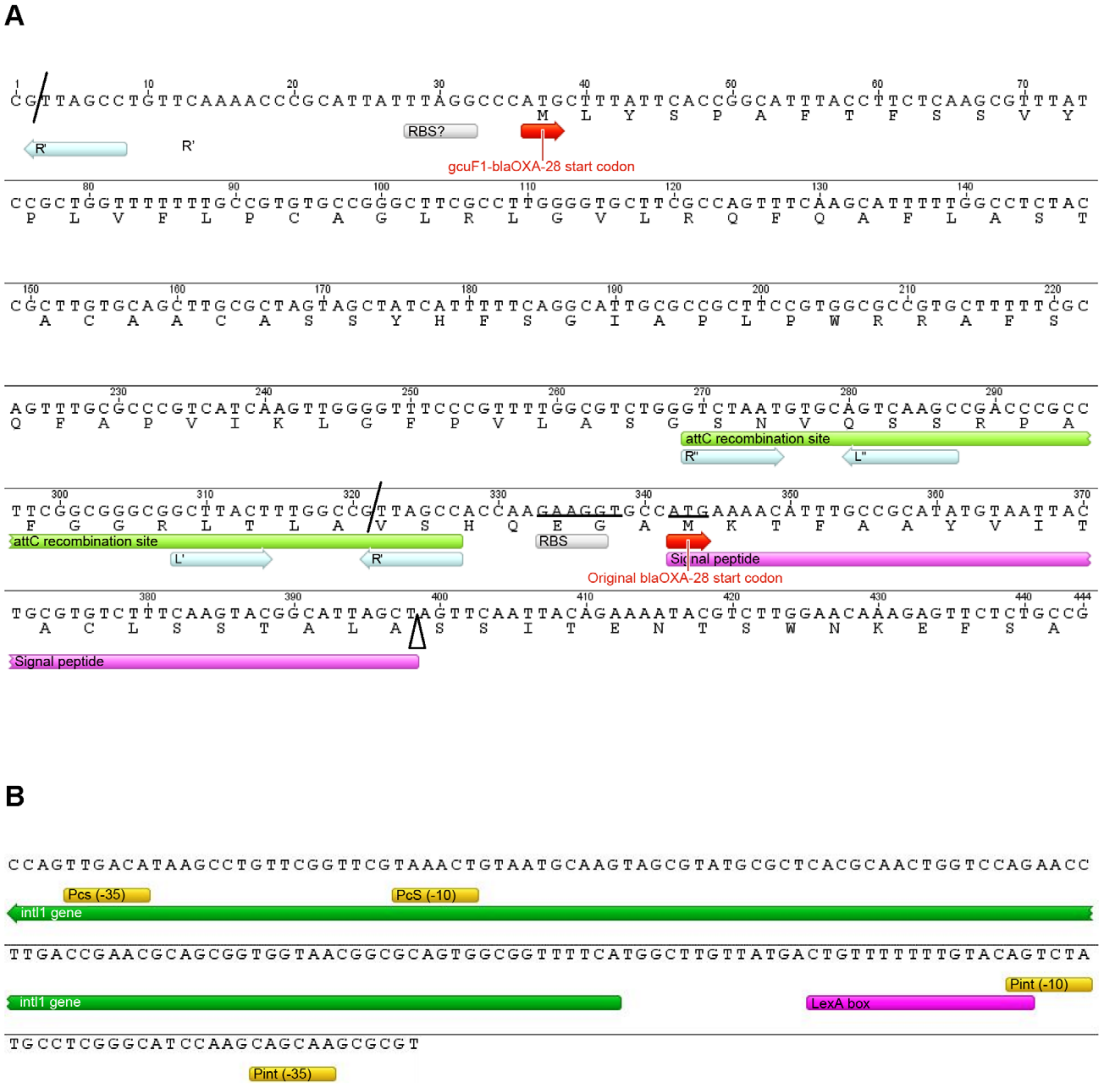
Details of the class 1 integron sequence from isolate S-*Pae*. (A) Nucleotide sequence of the 319-pb *gcuF1* cassette. Cassette boundaries are indicated by slashes. The putative ribosome binding site (RBS) and start codon of *gcuF1-bla*_OXA-28_ are indicated by a grey box and by a red arrow, respectively. The deduced amino acid sequence of the N-terminal portion of GCUF1-OXA-28 is designated in single-letter code below the nucleotide sequence. The *attC* recombination site of the *gcuF1* cassette is indicated in light green. The inverted repeats (R″, L″ and L′, R′) are indicated by light blue arrows. The putative original RBS and start codon of *bla*_OXA-28_ are indicated by a grey box and a red arrow, respectively. Mutated nucleotides are underlined. The signal peptide is indicated in pink and the cleavage site by an open triangle. (B) A putative LexA protein binding site was identified in the promoter region of the IntI1 integrase. The LexA protein binding site consensus (CTGTN_8_ACAG) is indicated in purple [7]. The putative σ^70^ promoter elements (−35 and −10) of PcS and Pint are shown in yellow and the *intI1* 5′ region is indicated in green.

S-*Pae* differed from R-*Pae*1 by a 10-fold lower amount of *bla*_OXA-28_ transcripts (Figure 2B) and by the presence of a 319-bp cassette, *gcuF1*, inserted immediately upstream of *bla*_OXA-28_ (Figure 1B). Using nested-PCR, we demonstrated the presence of free circular cassettes of *gcuF1* in S-*Pae* (Figure 3A, B), demonstrating that the recombination between its own *attC* site and the *aacA4 attC* site was occurring, though at extremely low level [2]. Computational analysis of the nucleotide sequence of the S-*Pae* integron predicted the translation of a new ORF, a fused protein consisting of *gcuF1* and *bla*_OXA-28_ (GCUF1-OXA-28). We were able to show that these two cassettes (*gcuF1* and *bla*_OXA-28_) could be transcribed in a single transcript. Hence, we could retrieve a specific amplicon after PCR amplification using cDNA prepared from S-*Pae* RNA as the matrix and with primers overlapping the junction between *gcuF1* and *bla*_OXA-28_ (Figure S3). The GCUF1-OXA-28 peptide (368 residues) was predicted to have a molecular weight of 40.1 kDa, compared with the 29.3-kDa native OXA-28 (266 residues). To confirm these data, both ORFs were expressed in *Escherichia coli* BL21 from plasmid pET-28a which adds an N-terminal polyHis tag. After purification, we found that their molecular weights estimated by SDS-PAGE were in full agreement with our predictions (Figure 1D). This protein contained the original 19-residue long signal peptide now misplaced between the GCUF1 and the OXA-28 domains at position 103–121 of the GCUF1-OXA-28 protein (Figure 1B and 4A). Since β-lactamases are periplasmic proteins and are produced as preproteins with an N-terminal peptide signal [20], one would expect that the misplacement of the signal peptide in the GCUF1-OXA-28 protein will abolish the periplasmic process of the β-lactamase. However, cellular production of this altered protein conferred a residual resistance to ceftazidime (MIC of ceftazidime, 8 µg/ml; *gcuF1*-*bla*_OXA-28_ in Figure 1C), suggesting the presence of an active and processed OXA-28 in the periplasm of the GCUF1-OXA28-producing isolate. To clarify this point, we cloned the *bla*_OXA-28_ and *gcuF1*-*bla*_OXA-28_ sequences into the broad host range vector pBTK27 to encode C-terminal His-tagged polypeptides that were expressed in the reference strain *P. aeruginosa* PA14Δ*ampC*. Western-blot analysis of periplasmic extracts of GCUF1-OXA28-producing bacteria revealed the presence of a reduced amount of processed periplasmic OXA-28 (Figure 1D), consistent with the lower resistance to ceftazidime when the *gcuF1* cassette is inserted upstream of the *bla*_OXA-28_ (Figure 1C). We used a directed mutagenesis approach to clarify the origin of periplasmic OXA-28 in S-*Pae* and determine whether it is due to the export processing of the fusion protein or to an internal translational initiation at the original OXA-28 start codon (Figure 4A). In *P. aeruginosa* PA14Δ*ampC* carrying a plasmid-borne *gcuF1-bla*_OXA-28_, the in-frame insertion of a stop codon just upstream of *bla*_OXA-28_ reduced the resistance level to ceftazidime down to 2 µg/ml. We also tested the effect of the in frame deletion of the *bla*_OXA-28_ ribosome binding site (GAAGGT), or its substitution by a sequence with no ribosome binding properties (CTCTCT). Finally, we tested the substitution of the ATG start codon with either a GTC or a GTG valine codons, which have no or weak translation initiation power [21]. None of these mutations led to a change in resistance level (Figure 1C). Hence, we confirmed that detected OXA-28 came entirely from the processing of the ORF2-OXA28 fusion protein, and that the inefficiency of its maturation was in part responsible for the low resistance level to ceftazidime. Additionally, the putative ribosome binding site of *gcuF1* (TTAGG) is predicted to have a poor translation initiation efficiency [22], [23] (Figure 4A), likely leading to reduced translation of *gcuF1-bla*_OXA-28_.

### SOS response induction by metronidazole treatment

In patient 1, the transition from S-*Pae* to R-*Pae*1 was observed after treatment with two antibiotics, ceftazidime and metronidazole, both known to activate the bacterial SOS response [12], [24]. Cassette expression in class 1 integrons can be controlled by two promoters, Pc and P2, which can exist under various forms [4]. P2 is created by the insertion of three guanines between the potential −35 and −10 regions, but this insertion also disrupts the LexA binding box (also called the SOS box) of the *intI1* promoter and abrogates the SOS control of *intI1* expression [4]. Analysis of the S-*Pae* class 1 integron revealed a functional LexA-binding box overlapping the −10 box of the *intI1* promoter [25], thus the P2 promoter is absent and the cassettes' expression only relies on the strong Pc promoter variant PcS (Figure 4B). It has been previously found that the encoded integrase IntI1 (IntI1_R32_N39_) displayed the second highest excision activity of the four known existing variants [4]. In agreement with this, using nested-PCR, we confirmed the presence of free circular *gcuF1* cassettes in S-*Pae* (Figure 3A, B), occurring through recombination between its own *attC_gcuF1_* site and the *aacA4 attC_aacA4_* site, as observed previously for a few other cassettes [2]. LexA is the transcriptional repressor that binds the SOS box sequences to silence transcription. RecA, once activated by the presence of abnormal single strand DNA produced by a variety of stimuli that includes antibiotic exposure, induces the LexA autoproteolysis and releases the transcriptional silencing driven by LexA binding to SOS boxes. We hypothesized that the excision of the *gcuF1* cassette by the integrase IntI1 and subsequent emergence of full resistance to ceftazidime in R-*Pae*1 was a result of the SOS response induced by antibiotic therapy in the patient.

**Figure 5:**
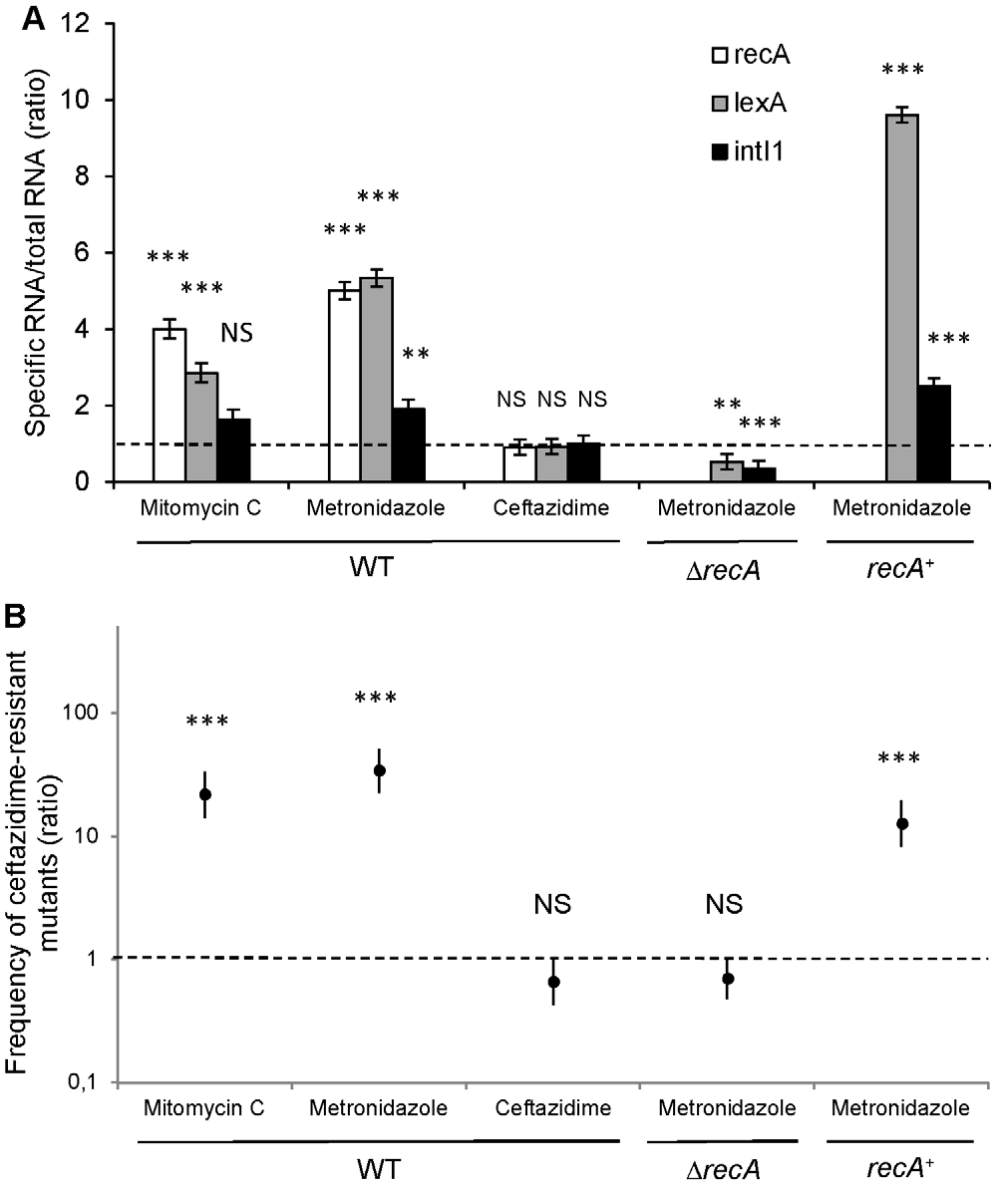
Metronidazole increases the emergence of ceftazidime-resistant mutants by inducing the SOS response. (A) Expression determination by RT-qPCR of *recA*, *lexA* and *intI1* with mitomycin C (a well-characterized SOS inducer used as a positive control [8]), metronidazole and ceftazidime in S-*Pae*Δ*ampC* (WT), S-*Pae*Δ*ampC*Δ*recA* (Δ*recA*) and S-*Pae*Δ*ampC*Δ*recA* complemented with *recA* (*recA* ^+^). Ratio of means ± SEM from at least 2 independent experiments in triplicate; two-sided Student's *t*-tests using 1.0 – dotted line – as comparator; NS, *P*>0.05; **, *P*<0.01; ***, *P*<0.001. (B) Frequency of emergence of ceftazidime-resistant mutant (by *gcuF1* cassette excision) in the same experimental conditions as for Figure 5A (ratios of geometric means of the excision rates ± SEM, N≥3; two-sided Student's *t*-tests using 1.0 – dotted line – as comparator; NS, *P*>0.05; ***, *P*<0.001).

We quantified the expression of SOS pathway genes *recA* and *lexA*, as well as the integrase encoding gene *intI1* by RT-qPCR after *in vitro* induction with metronidazole and ceftazidime. Our results indicated that *in vitro* exposure of S-*Pae*Δ*ampC* to the minimal inhibitory concentration of ceftazidime (2 µg/ml) neither triggered the SOS response (*recA* and *lexA* were not induced), nor enhanced the expression of the *intI1* gene (Figure 5A). We measured the frequency of ceftazidime-resistant mutant emergence by *gcuF1* cassette excision in the same experimental conditions. Consistent with the integrase expression data, we found that the *gcuF1* cassette excision frequency remained basal after exposure to ceftazidime (Figure 5B). On the contrary, *in vitro* exposure of S-*Pae*Δ*ampC* to therapeutic concentrations of metronidazole (50 µg/ml for *recA* wild-type strain and 25 µg/ml for *recA* mutants) triggered the SOS response, as indicated by the increased *recA* and *lexA* expression, and the increase in *intI1* expression, *gcuF1* cassette excision and a subsequent 34-fold enhancement of the frequency of emergence of ceftazidime-resistant mutants (Figure 5A, B). As shown with the results obtained in *recA*-deleted and *recA*-complemented strains, the effect of metronidazole on the integron rearrangement fully depended on the presence of *recA*, confirming the role of the SOS induction for the cassette rearrangement (Figure 5A, B). In mutants isolated both in patient 1 (R-*Pae*1) and *in vitro* (M-*Pae*), the *gcuF1* cassette excision provoked full *bla*_OXA-28_ expression and a massive increase in resistance to ceftazidime (Figure 1C).

## Discussion

In this study, we identified a new mechanism of modulation of antibiotic resistance in integrons. The positional regulation of gene cassette expression was already documented, but this was considered so far as only relying on the transcription attenuation process and on the lack of transcriptional coupling between genes carried in consecutive cassettes [6]. What we describe here is the presence of a genetic element, the *gcuF1* cassette, upstream of the integron-borne β-lactamase cassette *bla*_OXA-28_ which modulates the transcription, translation, and secretion of this enzyme, all at once. The poor ribosome-binding site found upstream of *gcuF1* (Figure 4A) [23] is likely responsible for the low production of the fusion protein detected, but is also likely responsible for the low level of *bla*_OXA-28_ mRNA. Indeed, it has been shown that a reduced ribosome binding to RBS can destabilize mRNA, which then becomes more vulnerable to endonucleolytic attack [26]. *GcuF1* shares 78% identity with integron-borne *orfD* gene cassettes of unknown function that are frequently found in clinical strains of *Pseudomonas* sp. and *Enterobacteriaceae* [1]. The insertion of *gcuF1* generates a fusion protein GCUF1-OXA-28 with a misplaced signal peptide between the GCUF1 and the OXA-28 domains. However, the GCUF1-OXA-28-producing bacteria still demonstrated residual resistance to ceftazidime, consistent with the presence of small amount of the processed OXA-28 in its periplasm. Using various mutants constructed in this aim, we established that the OXA-28 produced from *gcuF1-bla*_OXA-28_ was exclusively derived from cleavage at position 121 of the fusion protein GCUF1-OXA-28 (Figure 4A). These data confirm that a protein with a misplaced cleavable leader sequence (*i.e.* outside the N-terminus) can be exported, although less efficiently, into the periplasm [27].

We showed that the *gcuF1* cassette can be excised by the IntI1 integrase, leading to the production of a circularized cassette. The *gcuF1* cassette carries an *attC* site with an unusual R″ box, with a T instead of a C in last position, as in the large majority of integron cassettes (Figure 4A). The *gcuF1* closest relative is a cassette found in the *Acidovorax* sp. JS42 genome (GenBank accession number CP000539), which is 87% identical over the whole cassette sequence, but shows a CC at this precise position. Thus the substitution of this dinucleotide by a single T explains why the spacer between the R″ and L″ boxes is reduced to 4 nucleotides, instead of the usual 5 nucleotides in *gcuF1* (Figure 4A). The last base of R″ is normally pairing with the first base of the R′ box in the single strand recombinogenic form of the *attC* site [28]. Frumerie and colleagues tested all possible base pairs (C/G, A/T, G/C and T/A) at this position in the predicted annealed R″/R′, and found that all deeply decreased the recombination rate, by more than a hundred fold factor [29]. However the effect of single base substitutions at these positions is so far unknown, the observations made in our study suggest that substitution of the conserved C in R″ by a T does not abolish the *attC* recognition and recombination, but the effect of this mutation, as well as the one brought by the R″/L″ spacer reduction, on the rate of recombination needs to be established.

We found that excision of the *gcuF1* cassette from the original cassette array leads to increased resistance to ceftazidime (Figure 1C). As the expression of IntI1 is controlled by the SOS response, we surmised the antibiotic treatment given in first instance to this patient (ceftazidime and metronidazole) to be responsible for the SOS induction episode that ultimately led to the IntI1-mediated *gcuF1* deletion. We found that, in contrast to ceftazidime, *in vitro* exposure to therapeutic concentrations of metronidazole, an antimicrobial against which *P. aeruginosa* is naturally resistant, greatly enhanced the frequency of emergence of ceftazidime-resistant mutants. This phenotype is dependent on the excision of the *gcuF1* cassette that is fully dependent on the SOS response, as attested by the lack of excision in a *recA* mutant (Figure 5B). We speculate that in patient 1, metronidazole likely promoted the SOS-dependent transition from S-*Pae* to R-*Pae*1, which was further selected by ceftazidime therapy.

Interestingly, Cipriano Souza *et al.* showed that previous consumption of metronidazole was an independent risk factor for acquisition of multi-drug resistant *P. aeruginosa* by hospitalized patients [30]. Metronidazole and related 5-nitroimidazoles are redox-active prodrugs. Metronidazole is widely used to treat anaerobic bacteria infections, (*e.g. Clostridium difficile*), protozoa, and the microaerophilic *Helicobacter pylori* [31]. Bacterial nitroreductases, such as RdxA in *H. pylori*, catalyze the conversion of metronidazole to mutagenic products that directly interact with DNA bases [32], [33]. This causes DNA helix destabilization and single- and double-strand DNA breakage [34] that activate the SOS response [7], [24]. The effect of metronidazole in *P. aeruginosa*, in terms of DNA damage has still to be established, but one can speculate that the RdxA homolog in *P. aeruginosa* (PA5190 in the PAO1 genome, http://www.pseudomonas.com) could play a similar role in the metabolism of the metronidazole, and explain how this antibiotic triggers the SOS induction.

Our data suggest that SOS induction by antibiotics can result in the development of integron-based resistance *in vivo*. SOS also enhances the rate of mutations [7]. This is of particular concern in *P. aeruginosa* in which multidrug resistance mainly arise from chromosomal mutations [35]. More generally, it may lead to undesired changes in the behavior of bacteria and their faster adaptation to hostile environments. This is alarming because apart from metronidazole, other major classes of antibiotics (*e.g.* β-lactams, aminoglycosides, trimethoprim and fluoroquinolones) can trigger the bacterial SOS response [11]–[13].

The expression of horizontally acquired antibiotic resistance mechanisms is tightly regulated; this may reduce the biological cost associated with resistance expression and account for the dissemination of susceptible strains carrying hidden resistance determinants [36], [37]. Here, in S-*Pae*, expression of antibiotic resistance is silenced until antibiotic exposure triggers expression. This could represent an efficient evolutionary pathway for resistance determinants to be “switchable” and render bacteria fitness-neutral in the absence of antibiotic selection pressure [37]. Current policies for controlling the spread antibiotic resistance often rely on the detection of resistant bacteria, and on the assumption that resistance has a functional cost [38]. Future antibiotic restriction guidelines should consider the fact that resistance genes can spread latently in susceptible isolates with low biological cost.

In summary, we describe a reversible mechanism modulating an acquired antibiotic resistance in bacteria. The metronidazole-induced SOS response favored the emergence in a patient of bacteria highly resistant to ceftazidime that could then spread to twelve other patients which were under antibiotic pressure.

The suppression of the SOS response activation has been reported to enhance killing by antibiotics of *E. coli* and to increase survival of infected mice [39], [40]. Efforts have been made to identify small molecules and short peptides that inhibit RecA activity, although the absence of potential adverse effects on Rad51 (the human RecA homologue) needs to be demonstrated [41]–[43]. Our results suggest an adaptive role for the antibiotic-induced SOS response in bacterial genome rearrangement *in vivo* within humans. Altogether, this supports the hypothesis that inhibition of RecA is a plausible therapeutic adjuvant in combined therapy to reduce the capacity to generate antibiotic-resistant mutants.

## Materials and Methods

### Bacterial isolates

We identified a multidrug-resistant *P. aeruginosa* strain (R-*Pae*) in 13 patients hospitalized in the hematological ward of the University Hospital of Besançon (France) from March 2004 (Patient 1) to December 2009 (Patient 13). The genetic similarity of *P. aeruginosa* clinical isolates was investigated by pulsed field gel electrophoresis (PFGE; CHEF-DR III; Bio-Rad, Hercules, California) with the use of *Dra*I enzyme, as described elsewhere [44]. We retroactively analyzed the bacterial isolates of patient 1's early specimens. Twenty-eight days before pulmonary infection with R-*Pae*1, this patient was colonized with S-*Pae*, a clonally-related isolate that was more susceptible to β-lactams than R-*Pae*. Early and late sputums only contented S-*Pae* and R-*Pae*, respectively. In review of the patient record, patient 1 was treated with ceftazidime (4 g/day for 8 days) for *P. aeruginosa* and also with metronidazole (500 mg/day for 7 days) for infections by anaerobes prior to the isolation of R-*Pae*1. Oligonucleotides, bacterial strains and plasmids used for this study are detailed in the Tables S2, S3 and S4, respectively.

### Determination of the resistance level to antibiotics

The minimal inhibitory concentrations (MICs) of selected antibiotics were determined by the conventional Mueller-Hinton agar (MHA) dilution method, and interpreted according to CLSI (Clinical and Laboratory Standards Institute) guidelines [45]. The wild-type reference strain of *P. aeruginosa* PA14 was used as a control in susceptibility testing. MHA was supplemented with 1 mM of IPTG for strains carrying pBTK27 derivatives (Table S4).

### Real time quantitative RT-PCR (RT-qPCR)

Total RNA was isolated from cultures at an absorbance at 600 nm of 1.0 (or otherwise stated) using the Qiagen RNeasy protocol (Qiagen, Valencia, California). The RNA samples were treated with DNase (Turbo DNAse; Ambion, Austin, Texas) and further cleaned according to the manufacturer's protocol. Total RNA was quantified using the RiboGreen RNA Quantitation Kit (Molecular Probes, Carlsbad, California). Total RNA was reverse transcribed with Superscript III reverse transcriptase (Invitrogen, Carlsbad, California) as specified by the supplier. Quantitative PCR was performed on an Mx4000 Multiplex QPCR System (Stratagene, Santa Clara, California) using samples in triplicate with 25 ng of total RNA in a 20 µl reaction using SYBR Green PCR Master Mix (Applied Biosystems, Carlsbad, California) and specific primers for housekeeping gene *rpsL*, *bla*_OXA-28_, *ampC*, *recA*, *lexA*, and *intI1* (Table S2). PCR cycling conditions consisted of 95°C for 10 min, and 40 cycles of 95°C for 15 s, 60°C for 1 min. After each assay, a dissociation curve was run to confirm specificity of all PCR amplicons. The mRNA levels of *ampC* and *bla*_OXA-28_ were normalized to that of reference gene *rpsL* [46] and expressed as a ratio to the levels in the isolate PA14 (for *ampC*) or R-*Pae*1 (for *bla*_OXA-28_) in which the values were set at 1.00. For *recA*, *lexA*, and *intI1* genes, resulting *C*t values were converted to nanograms, normalized to total RNA and expressed as the average of triplicate samples.

### Evidence of circular forms of *gcuF1* cassette

We assessed the presence in S-*Pae* isolate of free circular forms of the *gcuF1* cassette. Total DNA from isolates R-*Pae*1 (without *gcuF1*, taken as a control) and S-*Pae* (with *gcuF1*) were PCR amplified with primers circ1 and circ2 (Figure 3A, Table S2). The purified PCR products were used as templates for a second nested PCR with primers circ3 and circ4. PCR products were visualized on an agarose gel. The PCR fragment obtained from S-*Pae* DNA was further sequenced to verify its specificity.

### Evidence of *gcuF1*-*bla*_OXA-28_ transcripts

To determine whether the DNA element *gcuF1*-*bla*_OXA-28_ could transcribe a functional transcript, we carried out RT-PCR reactions by using PCR primers overlapping the *gcuF1*-*bla*_OXA-28_ junction (overlap1 and overlap2, Table S1), and cDNA prepared from S-*Pae* RNA as the matrix (see above). The nucleotide sequence of the amplicon was determined to check for the specificity of the reaction.

### Deletion mutant construction

For the deletion of *ampC* from PA14 and S-*Pae*, approximately 1000-bp specific fragments upstream (with primers AmpCdel1F/AmpCdel1R) and downstream (with primers AmpCdel2F/AmpCdel2R) of *ampC* were PCR amplified from PA14 and S-*Pae* total DNAs, and used in an overlap extension reaction to create a single 2,000-bp product (Table S1). These products were cloned into Gateway-compatible gene replacement vector pEX18AmpGW [47], yielding the plasmids pDelAmpC-PA14 and pDelAmpC-SPae (Table S3), which were then transformed into *E. coli* DH5α.

For *recA* inactivation in S-*Pae*Δ*ampC*, 5′ (ca. 450-bp) and 3′ (ca. 530-bp) portions of *recA* were amplified separately with primer pairs RecAdel1F/RecAdel1R and RecAdel2F/RecAdel2R, respectively. The *tetA* gene was amplified from plasmid mini-CTX1 with primers TetF and TetR (Table S1). These three fragments were cloned simultaneously in a 4 way ligation in the *Eco*RI/*Hind*III sites of pEX18ap to yield plasmid pDelRecA (Table S3).

The plasmids for *ampC* or *recA* deletion were transferred into the recipient strains (PA14 or S-*Pae*Δ*ampC*) by triparental mating that included the donor strain *E. coli* DH5α with strain *E. coli* HB101 (containing helper plasmid pRK2013), followed by selection with irgasan (25 µg/ml) and carbenicillin (150 µg/ml for PA14, 500 µg/ml for S-*Pae*Δ*ampC*) and screening for *P aeruginosa* transconjugants with the deletion as previously described [48]. Deletion of the *ampC* and inactivation of *recA* were verified by PCR and sequencing.

### Construction of plasmids expressing *bla*_OXA-28_ and derivatives

The resistance level to ceftazidime conferred by the production of OXA-28, GCUF1-OXA-28, and their derivatives was assessed by cloning *gcuF1*-*bla*_OXA-28_ (PCRed from S-*Pae* with primers 1 and 3) and *bla*_OXA-28_ (PCRed from R-*Pae*1 with primers 2 and 3) sequences into the broad host range vector pBTK27. This yielded plasmids pBTK/gcuF1-oxa28 and pBTK/oxa28, respectively, encoding C-terminal polypeptides that were expressed in the reference strain *P. aeruginosa* PA14Δ*ampC* (Table S3). We used the plasmid pBTK/gcuF1-oxa28 as template for various mutageneses with a QuikChange kit (Stratagene). We inserted a TGA stop codon downstream *gcuF1* with mutagenic primers stop-F and stop-R, yielding plasmid pInsSTOP. We deleted in frame the GAAGGT sequence including the natural *bla*_OXA-28_ ribosome binding site (GAAGG) with mutagenic primers delRBS-F and delRBS-R, yielding plasmid pDelRBS, which encodes this GCUF1-OXA-28 variant missing amino acids E100 and G101. We also substituted the sequence harboring the *bla*_OXA-28_ ribosome binding site with a sequence with no translation initiation power (GAAGGT by CTCTCT) using mutagenic primers replRBS-F and replRBS-R, yielding plasmid pReplRBS. The ATG start codon from *bla*_OXA-28_ was substituted by GTC or by GTG with mutagenic primers (RepATG1-F/RepATG1-R and RepATG2-F/RepATG2-R, respectively) yielding plasmid pReplATG1 and pReplATG2, respectively, which encodes the M103V GCUF1-OXA-28 variant (Tables S2 and S4). All pBTK27-derivated plasmids were introduced into the reference strain *P. aeruginosa* PA14Δ*ampC* by triparental mating (see above) to assess the resistance to ceftazidime.

### OXA-28 and GCUF1-OXA-28 purification

To determine the size of the encoded proteins, *bla*_OXA-28_ (PCRed from R-*Pae*1 with primers 5 and 6) and *gcuF1*-*bla*_OXA-28_ sequences (PCRed from S-*Pae* with primers 4 and 6) were cloned into the pET-28a vector (Km^r^; Novagen-Merck, Darmstadt, Germany) at *Nhe*I/*Xho*I, yielding plasmids pET/oxa28 and pET/gcuF1-oxa28, respectively, encoding N-terminal His-tagged polypeptides. The cloned gene products were expressed in *E. coli* BL21(DE3) by IPTG induction (0.2 mM) to the exponentially growing cells (*A*_600_ of 0.8) and left overnight at 20°C with shaking. Bacteria were harvested and lysed using standard protocols. The lysates were applied on a 5 ml Ni-NTA column (Qiagen). His-tagged peptides were eluted with PBS supplemented with 250 mM imidazole. Eluted fractions were separated by 12% SDS-PAGE and transferred to nitrocellulose filters. Filters were hybridized with the His-detector Ni-HRP reagent (KPL) and the immune complexes were detected by the ECL-Plus chemiluminescent system (GE Healthcare, Buckinghamshire, United Kingdom).

### OXA-28 and GCUF1-OXA-28 subcellular localization

To assess the presence of His-tagged OXA-28 or GCUF1-OXA-28 in the periplasm, the plasmids pBTK/oxa28 or pBTK/gcuF1-oxa28 in *P. aeruginosa* PA14Δ*ampC* was induced by 1 mM IPTG for 4 h at 37°C. Periplasmic fractions were prepared by using Peripreps Periplasting kit (Epicentre Biotechnologies, Madison, Wisconsin) and analyzed by SDS-PAGE, transfer, and hybridization (see below). The raw integrated density of the blots was assessed using the ImageJ 1.44p software (National Institute of Health).

### Construction of plasmid expressing *recA*

We cloned *recA* (PCRed from S-*Pae* with primers RecA1 and RecA2) sequence into the broad host range vector pBTK27, yielding plasmid pBTK/recA (Tables S2 and S4). Plasmid pBTK/recA was introduced into the strain S-*Pae*Δ*ampC*Δ*recA* by triparental mating (see above).

### Frequency of emergence of ceftazidime-resistant mutants by *gcuF1* cassette excision

S-*Pae*Δ*ampC*, S-*Pae*Δ*ampC*Δ*recA*, and S-*Pae*Δ*ampC*Δ*recA* carrying pBTK/recA or pBTK27 plasmids were used to determine the frequency of emergence of ceftazidime-resistant mutants by *gcuF1* cassette excision. The gene *ampC* was deleted to avoid the emergence of resistant mutants overproducing this intrinsic β-lactamase. Bacteria were grown in LB broth (Luria-Bertani) overnight, then diluted 1∶250 and grown until OD_600_ = 0.3. Half of the cultures were then exposed to antibiotics (mitomycin C, 1 MIC for 1.5 h; ceftazidime, 1 MIC for 1 h; metronidazole, 1/40 MIC for 15 h). MICs of mitomycin C were 1.0/0.3 µg/ml, those of ceftazidime were 2/2 µg/ml and those of metronidazole were 2000/1000 µg/ml for S-*Pae* derivatives with or without the *recA* gene, respectively. Exposure to antibiotics in these conditions did not alter the growth rates of the bacteria. Metronidazole concentrations used for SOS response induction experiments (25 and 50 µg/ml) were in the range of those found in the plasma of treated patients [49]. Cultures were collected by centrifugation and washed twice with 0.9% NaCl. Appropriate dilutions in 0.9% NaCl were plated on MH plates with or without ceftazidime at 50 µg/ml. We checked the *gcuF1* excision by PCR in 133 clones growing on ceftazidime-containing media, obtained after exposure to mitomycin C, metronidazole and ceftazidime. Only one of them (0.8%) still displayed the *gcuF1* cassette. The *gcuF1* excision rate under antibiotic stress was then estimated as the number of ceftazidime-resistant colonies divided by the number of plated cells. The result was expressed as the ratio of the excision rates with and without antibiotic (for wild-type and Δ*recA* strains) and with or without *recA* (for *recA*-complemented strain). All assays were independently performed at least 3 times. All the samples were subjected to microscopic observation to ascertain that no filamented cells were present. We confirmed that the isolate S-*Pae* was not a hypermutator (see Text S1).

### Ethics statement

Approval and written informed consent from all subjects or their legally authorized representatives were obtained before study initiation. The study was approved by the ethical committee ‘Comité d'Etude Clinique’ of the Besançon hospital, Besançon, France.

### Statistical analysis

Student's *t*-tests were used to determine statistical significance for comparisons of gene expression (Figure 5A) and frequencies of emergence of ceftazidime-resistant mutants (by *gcuF1* excision; Figure 5B) with and without antibiotic (for wild-type and Δ*recA* strains) and with or without *recA* (for *recA*-complemented strain). Data were log transformed and variance estimates were pooled over similar experiments, resulting in pooled estimates of standard error of 0.24 with 65 degrees of freedom for the *t*-tests of Figure 2A and of 0.54 with 27 degrees of freedom for the *t*-tests of Figure 5B. Graphical examination supports the assumption of normality and homogeneous variation across experiments for the gene expression data Figure 5A and frequency the emergence of ceftazidime-resistant mutant data Figure 5B expressed on a log scale. The chosen significance threshold was 0.05 for all tests.

## Supporting Information

Figure S1 **The spread of a multi-drug resistant strain of R-** ***Pae*** **producing the extended-spectrum β-lactamase OXA-28.** Pulsed-field gel electrophoresis profiles of *Dra*I-digested DNA from *P. aeruginosa* isolates that were recovered from 13 patients in the Hematological ward of the University Hospital of Besançon (France) from March 2004 (isolate R-*Pae*1) to December 2009 (isolate R-*Pae*13). S-*Pae* was isolated from patient 1, 28 days before R-*Pae*1.
(TIFF)

Figure S2 **Double-disk synergy test with** ***P aeruginosa*** **isolate R-** ***Pae*** **1 producing the extended-spectrum β-lactamase OXA-28.** Diffusion test was performed on Mueller-Hinton agar [14]. Synergies were observed between disks containing the substrates cefepime (30 µg in the FEP disk) or ceftazidime (30 µg in CAZ disk) and (A) the oxacillinase inhibitors clavulanate (10 µg in the amoxicillin/clavulanate AMC disk) or (B) imipenem (10 µg in the IMP disk). Such synergies are usually noticed with *P aeruginosa* strains producing class A extended-spectrum β-lactamases and class D extended-spectrum oxacillinases [14].
(TIFF)

Figure S3 **The** ***gcuF1*** **-** ***bla*** **_OXA-28_ element allows the transcription of a single transcript.** Electrophoresis on 1% agarose of PCR products using primers overlapping the junction *gcuF1*-*bla*_OXA-28_ (overlap 1 and overlap2, see Table S1). Templates were as follows: genomic DNA of R-*Pae* (1) and S-*Pae* (2), RNA extract of S-*Pae* (3), cDNA obtained from S-*Pae* RNA (4), water (5). MW: Molecular weight (1 kb band is indicated).
(TIFF)

Table S1 **Resistance levels to antibiotics in the studied isolates of** ***P. aeruginosa*** **.**
(DOC)

Table S2 **List of primers used in this study.**
(DOC)

Table S3 **List of bacterial strains used in this study.**
(DOC)

Table S4 **List of plasmids used in this study.**
(DOC)

Text S1 **Identification of additional non-enzymatic resistance mechanisms to β-lactams, and determination of the hypermutator phenotype of S-** ***Pae*** **.**
(DOC)
