# Supplementary figures and images for: Evidence for Induction of Integron-Based Antibiotic Resistance by the SOS Response in a Clinical Setting
Source: PLoS Pathog. 2012 Jun 14;8(6):e1002778. doi: 10.1371/journal.ppat.1002778 (PMC3375312; doi:10.1371/journal.ppat.1002778)

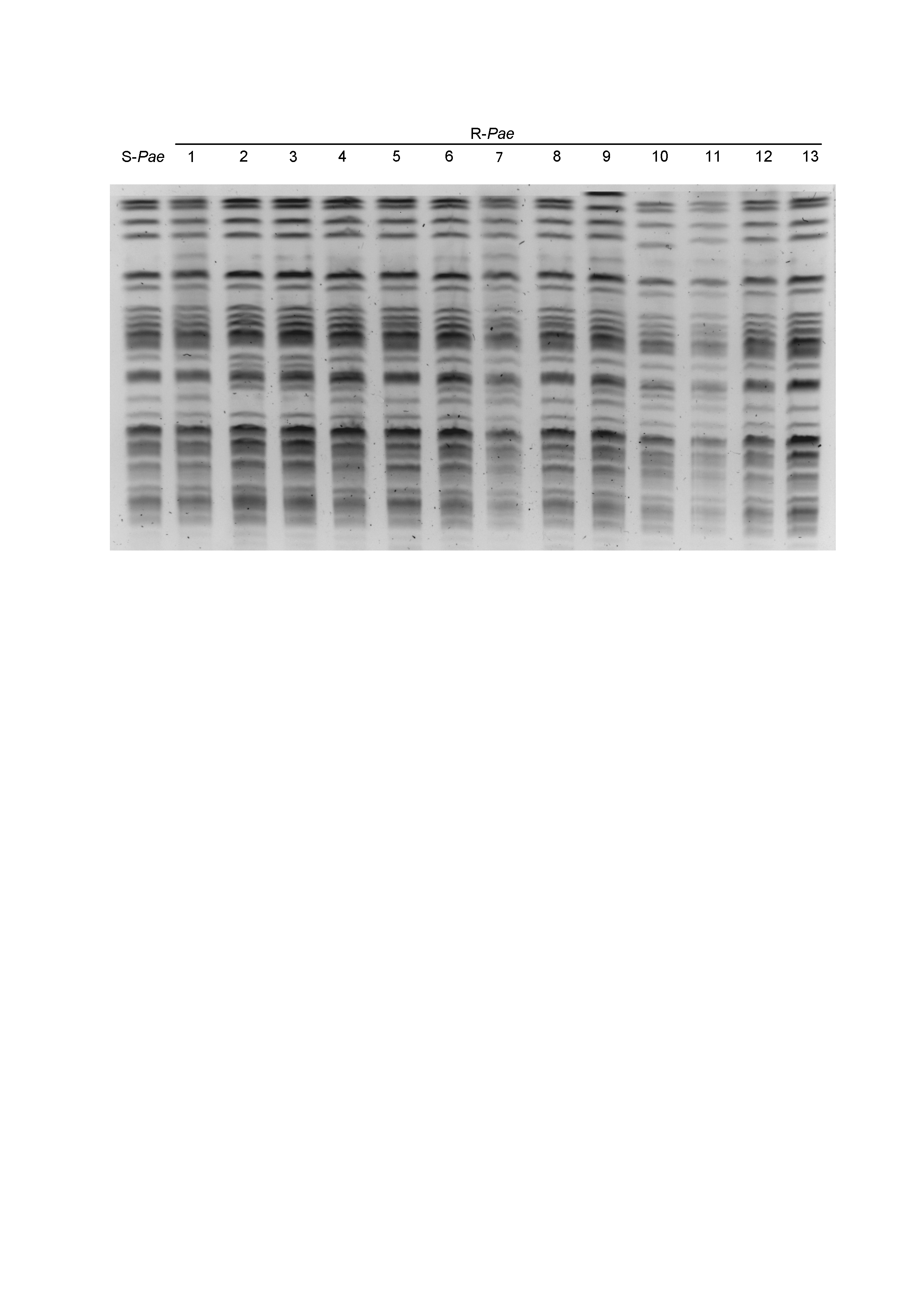

Supplement: Figure S1 — The spread of a multi-drug resistant strain of R- Pae producing the extended-spectrum β-lactamase OXA-28. Pulsed-field gel electrophoresis profiles of DraI-digested DNA from P. aeruginosa isolates that were recovered from 13 patients in the Hematological ward of the University Hospital of Besançon (France) from March 2004 (isolate R-Pae1) to December 2009 (isolate R-Pae13). S-Pae was isolated from patient 1, 28 days before R-Pae1. (TIFF) [file ppat.1002778.s001.tif]

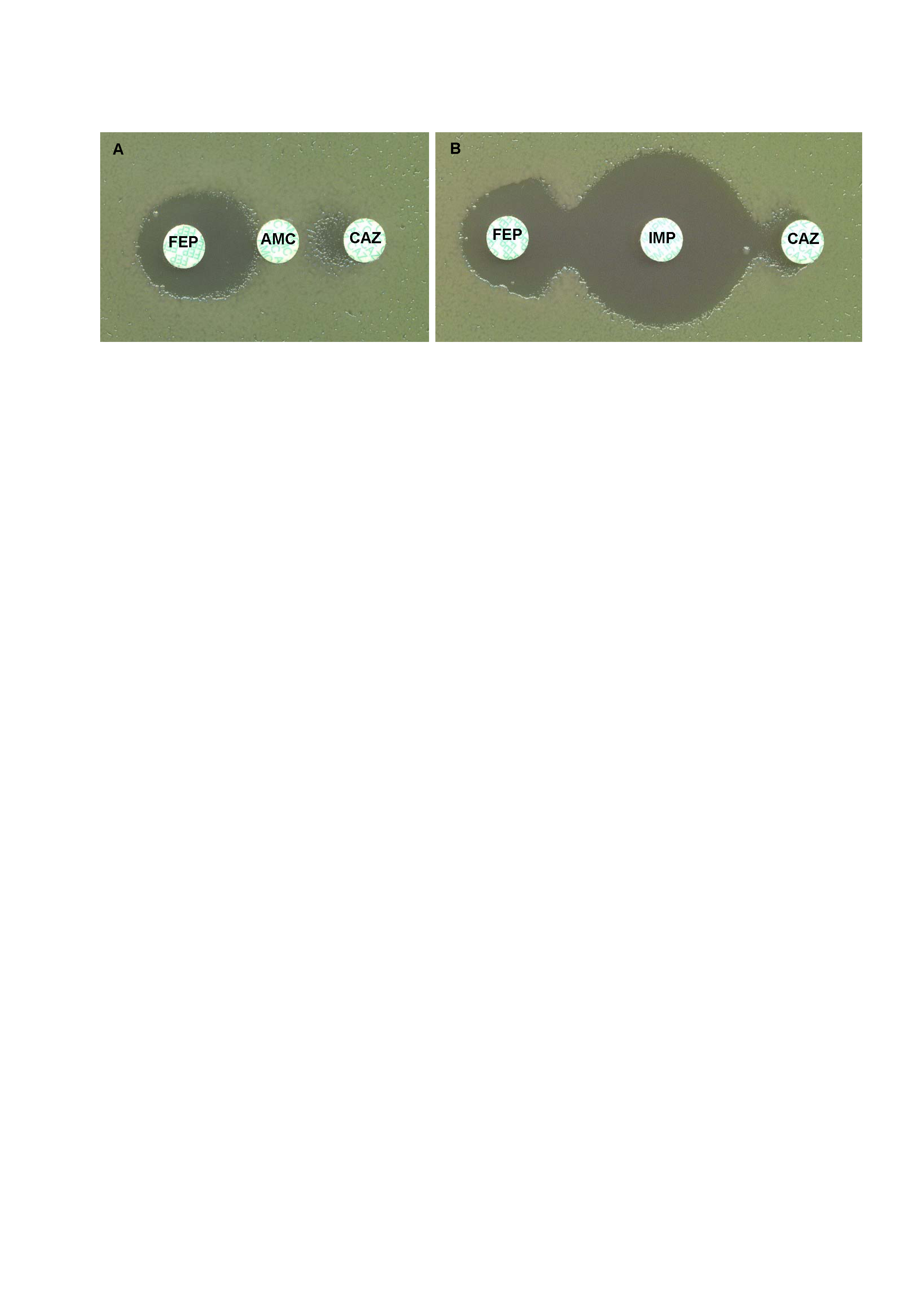

Supplement: Figure S2 — Double-disk synergy test with P aeruginosa isolate R- Pae 1 producing the extended-spectrum β-lactamase OXA-28. Diffusion test was performed on Mueller-Hinton agar [14]. Synergies were observed between disks containing the substrates cefepime (30 µg in the FEP disk) or ceftazidime (30 µg in CAZ disk) and (A) the oxacillinase inhibitors clavulanate (10 µg in the amoxicillin/clavulanate AMC disk) or (B) imipenem (10 µg in the IMP disk). Such synergies are usually noticed with P aeruginosa strains producing class A extended-spectrum β-lactamases and class D extended-spectrum oxacillinases [14]. (TIFF) [file ppat.1002778.s002.tif]

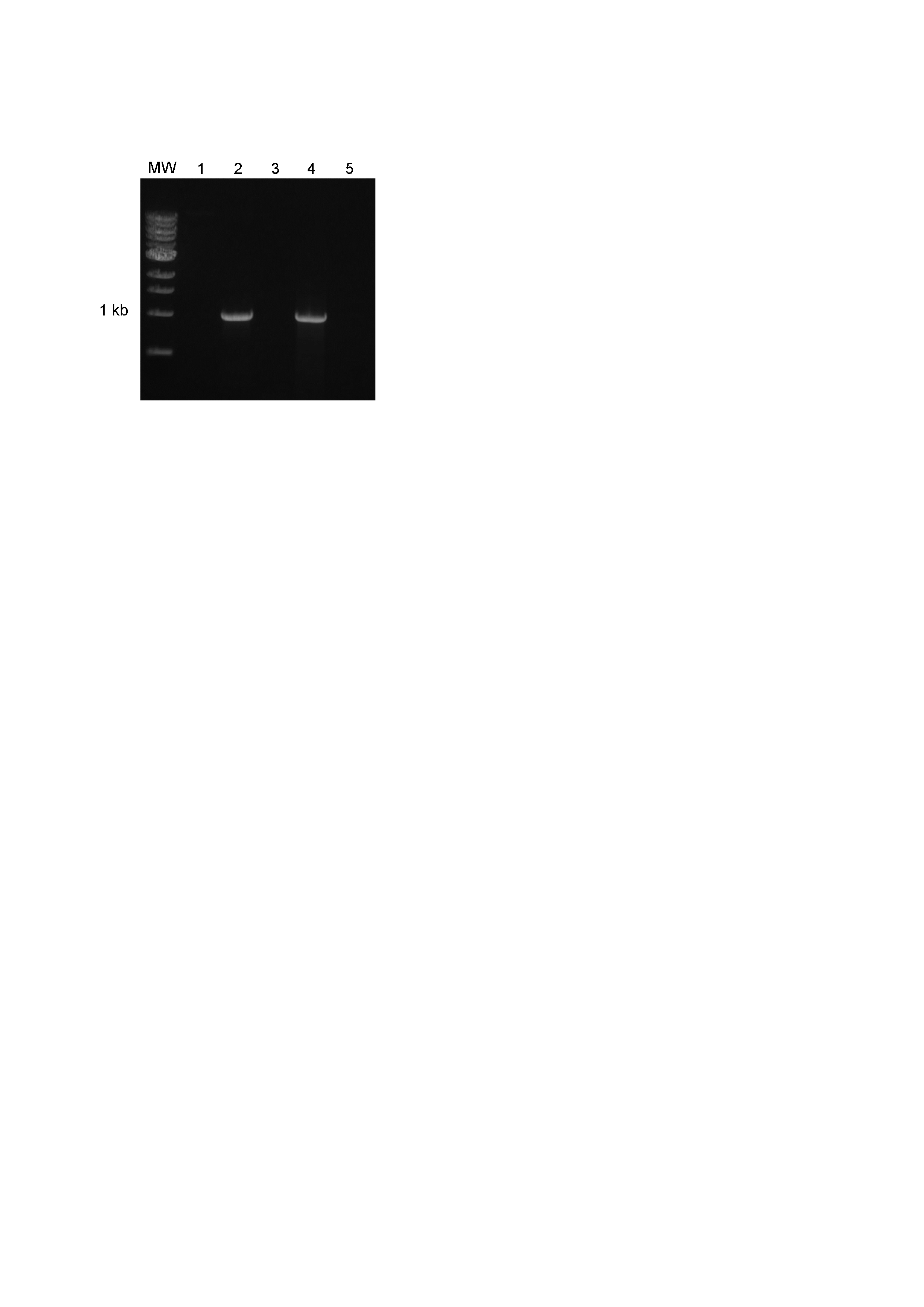

Supplement: Figure S3 — The gcuF1 - bla OXA-28 element allows the transcription of a single transcript. Electrophoresis on 1% agarose of PCR products using primers overlapping the junction gcuF1-bla OXA-28 (overlap 1 and overlap2, see Table S1). Templates were as follows: genomic DNA of R-Pae (1) and S-Pae (2), RNA extract of S-Pae (3), cDNA obtained from S-Pae RNA (4), water (5). MW: Molecular weight (1 kb band is indicated). (TIFF) [file ppat.1002778.s003.tif]
