# Supplementary material for: Evidence for Induction of Integron-Based Antibiotic Resistance by the SOS Response in a Clinical Setting
Source: PLoS Pathog. 2012 Jun 14;8(6):e1002778. doi: 10.1371/journal.ppat.1002778 (PMC3375312; doi:10.1371/journal.ppat.1002778)
Supplement: Table S1 — Resistance levels to antibiotics in the studied isolates of P. aeruginosa . (DOC) [file ppat.1002778.s004.doc]

**Table S1**

| Strain or isolate | |  | **MICs (µg/ml)^a^** | | | | | | | | |
| --- | --- | --- | --- | --- | --- | --- | --- | --- | --- | --- | --- |
| Name | Characteristic or patient(s) of origin | **Produced protein** | **CAZ** | **FEP** | **ATM** | **IMP** | **GEN** | **AMK** | **TOB** | **CIP** |  |
| PA14 | WT reference strain | - | **1** | **2** | **4** | **1** | **1** | **2** | **1** | **0.125** |  |
| S-*Pae* | Patient 1 | GCUF1-OXA-28 | **4** | **8** | **8** | 8 | 64 | **8** | 32 | 128 |  |
| M-*Pae* | Ceftazidime *in vitro* mutant from S-*Pae***^b^** | OXA-28 | 256 | nd**^c^** | nd | nd | nd | nd | nd | nd |  |
| R-*Pae*1 | Patient 1 | OXA-28 | 256 | 16 | 16 | 8 | 64 | **8** | 32 | 128 |  |
| R-*Pae*1 to 13 (*n*=12) | Patients 2 to 13 | OXA-28 | 256**^d^** | 32**^d^** | 32**^d^** | **4^d^** | 128**^d^** | **16^d^** | 32**^d^** | 128**^d^** |  |

**Table S1: Resistance levels to antibiotics in the studied isolates of *P. aeruginosa*.**

**^a^** Means of the MICs (Minimal inhibitory concentrations) are given (*n* ≥ 3, SEM are all equal to 0). Bolded MICs are within the susceptibility range. CAZ (ceftazidime), FEP (cefepime), ATM (aztreonam), IMP (imipenem), GEN (gentamicin), AMK (amikacin), TOB (tobramycin), CIP (ciprofloxacin).

**^b^** M-*Pae* is a mutant deriving from S-*Pae* and obtained *in vitro* after selection on ceftazidime 50 µg/mL. It lacks *gcuF1* and display an integron structure strictly identical to that of R-*Pae*.

**^c^** nd, not determined.

**^d^** Modal MICs are indicated
