# Supplementary material for: Evidence for Induction of Integron-Based Antibiotic Resistance by the SOS Response in a Clinical Setting
Source: PLoS Pathog. 2012 Jun 14;8(6):e1002778. doi: 10.1371/journal.ppat.1002778 (PMC3375312; doi:10.1371/journal.ppat.1002778)
Supplement: Table S2 — List of primers used in this study. (DOC) [file ppat.1002778.s005.doc]

**Table S2: List** of primers used in this study

| **Name** | **Sequence** | **Reference** |
| --- | --- | --- |
| OXA-10A | 5’**-** TCAACAAATCGCCAGAGAAG -3’ | [1] |
| OXA-10B | 5’**-** TCCCACACCAGAAAAACCAG -3’ | [1] |
| 5'-CS | 5’**-** GGCATCCAAGCAGCAAG -3’ | [2] |
| 3'‑CS | 5’**-** AAGCAGACTTGACCTGA -3’ | [2] |
| RPSL3 | 5’**-** GCAACTATCAACCAGCTGGTG -3’ | [3] |
| RPSL5 | 5’**-** GCTGTGCTCTTGCAGGTTGTG -3’ | [3] |
| AMPC-RT1 | 5’**-** CGGCTCGGTGAGCAAGACCTTC -3’ | [3] |
| AMPC-RT2 | 5’**-** AGTCGCGGATCTGTGCCTGGTC -3’ | [3] |
| OXA28-RT1 | 5’**-** CAATTATCGGCCTAGAAACTGG -3’ | This work |
| OXA28-RT2 | 5’**-** CTTGTATTGCCCCTCTTAAGCTC -3’ | This work |
| RECA-RT1 | 5’**-** CGCGGCCCTGGGACAGATCG -3’ | This work |
| RECA-RT2 | 5’**-** GATGCCGAGGGCGATGTCCAG -3’ | This work |
| LEXA-RT1 | 5’**-** CTCCTTCATCAAGCGCTGCCTGG -3’ | This work |
| LEXA-RT2 | 5’**-** GTTCGGCGACTTGAAGCCGAGTT -3’ | This work |
| INTI1-RT1 | 5’**-** TTCATCCGTTTCCACGGTGTGCG -3’ | This work |
| INTI1-RT2 | 5’**-** GAGACCGAAACCTTGCGCTCGTT -3’ | This work |
| Overlap1 | 5’- TCACCGGCATTTACCTTCTC -3’ | This work |
| Overlap2 | 5’- TGTTCCCTTCTCAACCCAAC -3’ | This work |
| 5 | 5’**-** CACCCC**GCTAGC**AAAACATTTGCCGCATATGTAATTAC -3’ | This work |
| 6 | 5’**-** TATATA**CTCGAG**TTGTTAGCCACCAATGATGCCCTC -3’ | This work |
| 4 | 5’**-** CACCCC**GCTAGC**CTTTATTCACCGGCATTTACCTTC -3’ | This work |
| circ1 | 5’**-** AAAAATGCTTGAAACTGGCG -3’ | This work |
| circ2 | 5’**-** TGTGCAGCTTGCGCTAGTAG -3’ | This work |
| circ3 | 5’**-** GAGAAGGTAAATGCCGGTGA -3’ | This work |
| circ4 | 5’**-** CTAATGTGCAGTCAAGCCGA -3’ | This work |
| AmpCdel1F | 5’**-** CACCGG**GGTACC**CAGCAACCACTCCCTGAACG -3’ | This work |
| AmpCdel2F | 5’**-** ACGCCAATCCTCATGCGCGATTGAGGCGCGCTCGCGAGG -3’ | This work |
| AmpCdel1R | 5’**-** CCTCGCGAGCGCGCCTCAATCGCGCATGAGGATTGGCGT -3’ | This work |
| AmpCdel2R | 5’**-** ATATAT**GGATCC**AGCTGATCGTCCAGGTGCT -3’ | This work |
| RecAdel1F | 5’**-** ATAT**GAATTC**GCGGCCCTGGGACAGATCG -3’ | This work |
| RecAdel2F | 5’**-** ATAT**GAGCTC**CCTGATGTCCCAGGCGCTGC -3’ | This work |
| RecAdel1R | 5’**-** ATAT**GGATCC**CGTCGCCCATCTCGCCTTCG -3’ | This work |
| RecAdel2R | 5’**-** ATAT**AAGCTT**GGCTTCGGCGTCAGCCACTT -3’ | This work |
| TetF | 5’**-** ATAT**GGATCC**GCTCACTGACTCGCTGCGCT -3’ | This work |
| TetR | 5’**-** ATAT**GAGCTC**TGCGCATTCACAGTTCTCCGCA -3’ | This work |
| 1 | 5’**-** CACCCC**GGTACC**TAAGAAGGAGATATCATATGCTTTATTCACCGGCATTTACCTTCTCAAGC -3’ | This work |
| 3 | 5’**-** ATATAT**AAGCTT**TTAATGATGATGATGATGATGGCCACCAATGATGCCCTCACTTGC -3’ | This work |
| 2 | 5’**-** CACCCC**GGTACC**TAAGAAGGAGATATCATATGAAAACATTTGCCGCATATGTAATTACTGCGTGT -3’ | This work |
| Stop-F | 5’**-** ttggccgttagccactgacaagaaggtgccatg -3’ | This work |
| Stop-R | 5’**-** catggcaccttcttgtcagtggctaacggccaa -3’ | This work |
| DelRBS-F | 5’**-** tactttggccgttagccaccaagccatgaaaacat -3’ | This work |
| DelRBS-R | 5’**-** atgttttcatggcttggtggctaacggccaaagta -3’ | This work |
| ReplRBS-F | 5’- ggcggcttactttggccgttagccaccaactctctgccatgaaaacatttgc -3’ | This work |
| ReplRBS-R | 5’- gcaaatgttttcatggcagagagttggtggctaacggccaaagtaagccgcc -3’ | This work |
| RepATG1-F | 5’**-** tagccaccaagaaggtgccgtcaaaacatttgccgcatatg -3’ | This work |
| RepATG1-R | 5’**-** catatgcggcaaatgttttgacggcaccttcttggtggcta -3’ | This work |
| RepATG2-F | 5’**-** ccaccaagaaggtgccgtgaaaacatttgccgc -3’ | This work |
| RepATG2-R | 5’- gcggcaaatgttttcacggcaccttcttggtgg-3’ | This work |
| RecA1 | 5’**-** CACCCC**GAGCTC**TAATCTTCCCGTCGGGGGTAGGC -3’ | This work |
| RecA2 | 5’**-** ATATAT**AAGCTT**TTACGGGCGTATCGAGCACGGTC -3’ | This work |

Restriction sites are in bold cases.

**References**

1. Bert F, Branger C, Lambert-Zechovsky N (2002) Identification of PSE and OXA β-lactamase genes in *Pseudomonas aeruginosa* using PCR-restriction fragment length polymorphism. J Antimicrob Chemother 50: 11-18.

2. Levesque C, Piche L, Larose C, Roy P (1995) PCR mapping of integrons reveals several novel combinations of resistance genes. Antimicrob Agents Chemother 39: 185-191.

3. Dumas J-L, van Delden C, Perron K, Köhler T (2006) Analysis of antibiotic resistance gene expression in *Pseudomonas aeruginosa* by quantitative real-time-PCR. FEMS Microbiol Lett 254: 217-225.
