# Supplementary material for: Evidence for Induction of Integron-Based Antibiotic Resistance by the SOS Response in a Clinical Setting
Source: PLoS Pathog. 2012 Jun 14;8(6):e1002778. doi: 10.1371/journal.ppat.1002778 (PMC3375312; doi:10.1371/journal.ppat.1002778)
Supplement: Table S3 — List of bacterial strains used in this study. (DOC) [file ppat.1002778.s006.doc]

**Table S3:** List of bacterial strains used in this study

| **Name** | **Relevant phenotype** | **Reference** |
| --- | --- | --- |
| S-*Pae* | Clinical *P aeruginosa* (from patient 1) susceptible to ceftazidime, expressing *gcuF1*-*bla_OXA-28_* | This work |
| R-*Pae*1 | Clinical *P aeruginosa* (from patient 1) resistant to ceftazidime, expressing *bla_OXA-28_* | This work |
| M-*Pae* | Ceftazidime-resistant mutants from S-*Pae* (selected on ceftazidime 50 µg/mL) expressing *bla_OXA-28_* | This work |
| R-*Pae*2 to 13 | Clinical *P aeruginosa* (from patients 2 to 13) resistant to ceftazidime expressing *bla_OXA-28_* | This work |
| PA14 | Wild type reference strain of *P aeruginosa*, UCBPP-PA14, human isolate | [1] |
| PAO1 | Wild type reference strain of *P aeruginosa*, human isolate | [2] |
| PA14Δ*ampC* | *P aeruginosa* PA14Δ*ampC* | This work |
| S-*Pae*Δ*ampC* | *P aeruginosa* S-*Pae*Δ*ampC* | This work |
| S-*Pae*Δ*ampC*Δ*recA* | *P aeruginosa* S-*Pae*Δ*ampC*Δ*recA* | This work |
| *E.* *coli* DH5α | *supE44* *endA1* *hsdR17*(rK^-^ mK^+^) *thi-1* *recA1* Δ(*argF*-*lacZYA*)*U169* Φ 80d*lacZ*ΔM15 *phoA* *gyrA96* *relA1* *deoR* λ^-^ | Gibco-BRL |
| *E. coli* BL21(DE3) | Expession host, F – *ompT hsdSB(rB– mB–) gal dcm* (DE3) | Novagen-Merck |

**References**

1. Lee DG, Urbach JM, Wu G, Liberati NT, Feinbaum RL, et al. (2006) Genomic analysis reveals that *Pseudomonas aeruginosa* virulence is combinatorial. Genome Biol 7: R90.

2. Stover CK, Pham XQ, Erwin AL, Mizoguchi SD, Warrener P, et al. (2000) Complete genome sequence of *Pseudomonas aeruginosa* PAO1, an opportunistic pathogen. Nature 406: 959-964.
