# Supplementary material for: Evidence for Induction of Integron-Based Antibiotic Resistance by the SOS Response in a Clinical Setting
Source: PLoS Pathog. 2012 Jun 14;8(6):e1002778. doi: 10.1371/journal.ppat.1002778 (PMC3375312; doi:10.1371/journal.ppat.1002778)
Supplement: Table S4 — List of plasmids used in this study. (DOC) [file ppat.1002778.s007.doc]

**Table S4:** List of plasmids used in this study

| **Name** | **Description** | **Reference** |
| --- | --- | --- |
| pET-28a | Expression vector (His Tag) Kan^r^ | Novagen-Merck |
| pET/oxa28 | *Nhe*I-*Xho*I fragment from R-*Pae*1 cloned into pET-28a | This work |
| pET/gcuF1-oxa28 | *Nhe*I-*Xho*I fragment from S-*Pae* cloned into pET-28a | This work |
| pEX18ap-GW | Gateway suicide destination vector; Amp^r^ | [1] |
| pDelAmpC-PA14 | pEX18ApGW with SOE fragment for deletion of the *P aeruginosa* PA14 *ampC* | This work |
| pDelAmpC-SPae | pEX18ApGW with SOE fragment for deletion of the *P aeruginosa* S-*Pae* *ampC* | This work |
| Mini-CTX1 | Source for *tetA* gene | [2] |
| pDelRecA | *Eco*RI-*Bam*HI fragment containing the 5’ portion of *recA*, *Bam*HI-*Sac*I fragment containing *tetA*, *Sac*I-*Hin*dIII fragment containing the 3’ portion of *recA* cloned into *Eco*RI-*Hin*dIII sites of pEX18Ap | This work |
| pRK2013 | ColE1 Tra1 Mob1 (pRK2) Km^r^ | [3] |
| pBTK27 | Broad host range expression vector derivated from pMMB67EH Gateway with an additional *lac* repressor *lacI^Q^* | [4] |
| pBTK/gcuF1-oxa28 | *Kpn*I*-Hind*III fragment from S-*Pae* cloned into pBTK27 | This work |
| pBTK/oxa28 | *Kpn*I*-Hind*III fragment from R-*Pae*1 cloned into pBTK27 | This work |
| pInsSTOP | pBTK/gcuF1-oxa28 (TGA stop codon insertion downstream *gcuF1*) | This work |
| pDelRBS | pBTK/gcuF1-oxa28 (*bla*_OXA-28_ ribosome binding site deletion) | This work |
| pReplRBS | pBTK/gcuF1-oxa28 (substitution of the sequence GAAGGT including *bla*_OXA-28_ ribosome binding site by CTCTCT) | This work |
| pReplATG1 | pBTK/gcuF1-oxa28 (*bla*_OXA-28_ start codon ATG→GTC) | This work |
| pReplATG2 | pBTK/gcuF1-oxa28 (*bla*_OXA-28_ start codon ATG→GTG) | This work |
| pBTK/RecA | *Sac*I-*Hind*III fragment from S-*Pae* cloned into pBTK27 | This work |

**References**

1. Choi KH, Schweizer HP (2005) An improved method for rapid generation of unmarked *Pseudomonas aeruginosa* deletion mutants. BMC Microbiol 5: 30.

2. Hoang TT, Kutchma AJ, Becher A, Schweizer HP (2000) Integration-proficient plasmids for *Pseudomonas aeruginosa*: site-specific integration and use for engineering of reporter and expression strains. Plasmid 43: 59-72.

3. Figurski DH, Helinski DR (1979) Replication of an origin-containing derivative of plasmid RK2 dependent on a plasmid function provided in trans. Proc Natl Acad Sci USA 76: 1648-1652.

4. Kulasakara H, Lee V, Brencic A, Liberati N, Urbach J, et al. (2006) Analysis of Pseudomonas aeruginosa diguanylate cyclases and phosphodiesterases reveals a role for bis-(3'-5')-cyclic-GMP in virulence. Proc Natl Acad Sci U S A 103: 2839-2844.
