# Supplementary material for: Evidence for Induction of Integron-Based Antibiotic Resistance by the SOS Response in a Clinical Setting
Source: PLoS Pathog. 2012 Jun 14;8(6):e1002778. doi: 10.1371/journal.ppat.1002778 (PMC3375312; doi:10.1371/journal.ppat.1002778)
Supplement: Text S1 — Identification of additional non-enzymatic resistance mechanisms to β-lactams, and determination of the hypermutator phenotype of S- Pae . (DOC) [file ppat.1002778.s008.doc]

**Appendix**

**Identification of additional non-enzymatic resistance mechanisms to β-lactams.** Using RT-qPCR methods already described [1], we found that the 14 clonal isolates (S‑*Pae*, R-*Pae*1 to 13) overexpressed the operons *mexAB-oprM* and *mexXY* compared to the wild-type strain PA14. These two operons encode the efflux pumps MexAB-OprM and MexXY, respectively. In all the isolates, the overexpression of *mexAB-oprM* was due to a mutation in the repressor *mexR* (Lys_44_Met) and that of *mexXY* due to a mutation in the repressor *mexZ* (Val_48_Ala). The overproduction of these two efflux pumps is responsible for a low level resistance to ticarcillin and aztreonam (MexAB-OprM) and to cefepime (MexAB-OprM and MexXY). Using the same method, we found that all the isolates underexpressed *oprD* compared to PA14. This gene encodes a carbapenem-specific porin, which underexpression is responsible for the specific resistance to carbapenems (*e.g.* imipenem). None of these three resistance mechanisms alter the susceptibility of the bacteria to ceftazidime.

**Determination of the hypermutator phenotype of S-*Pae*.** We checked whether the isolate S-*Pae* was a hypermutator, using the procedure previously described [2]. The frequency of emergence of rifampin-resistant mutants from S‑*Pae* isolate (4.5 10^-7^ ± 1.8 10^-7^) was not significant (*P* = 1.0, *n* = 3, two-sided Wilcoxon rank-sum test) than from the reference strain *P. aeruginosa* PAO1 (6.5 10^-7^ ± 2.1 10^-7^).

**References**

1. Dumas J-L, van Delden C, Perron K, Köhler T (2006) Analysis of antibiotic resistance gene expression in *Pseudomonas aeruginosa* by quantitative real-time-PCR. FEMS Microbiol Lett 254: 217-225.

2. Oliver A, Canton R, Campo P, Baquero F, Blazquez J (2000) High frequency of hypermutable *Pseudomonas aeruginosa* in cystic fibrosis lung infection. Science 288: 1251-1254.
